# Cold-Pressing as an Energy-Saving Dewatering Pretreatment for the Valorization of Vegetable Processing By-Products

**DOI:** 10.3390/foods15193481

**Published:** 2026-09-29

**Authors:** Chiara Russo, Matteo Alessandro Del Nobile, Amalia Conte

**Affiliations:** 1 Department of Economics, University of Foggia, Via Romolo Caggese, 71122 Foggia, Italy; chiara.russo@unifg.it; 2 Department of Economics, Management and Territory, University of Foggia, Via Alberto da Zara 11, 71122 Foggia, Italy; 3 Department of Humanistic Studies, Letters, Cultural Heritage, Educational Sciences, University of Foggia, Via Arpi. 176, 71121 Foggia, Italy; amalia.conte@unifg.it

**Keywords:** mechanical dewatering, cold-press dewatering, by-products, drying, recycling

## Abstract

In this study, an integrated process combining cold-press dewatering and thermal dehydration was proposed to reduce the energy required for powder production while simultaneously recovering a liquid fraction enriched in soluble bioactive compounds. Asparagus, broccoli and artichoke by-products were selected as representative vegetable matrices because of their markedly different anatomical organization and physicochemical characteristics, allowing the robustness of the proposed approach to be evaluated across heterogeneous biomasses. By-products and the corresponding solid residues obtained after cold-press dewatering were dehydrated under identical conditions and subsequently milled into powders. Moisture content, drying kinetics and nutritional quality of whole by-products and corresponding residues were assessed and compared. The specific energy consumption and the percentage increase in specific energy consumption associated with the omission of the cold-press dewatering step were also calculated using an approach already available in the literature. The preliminary dewatering step significantly reduced the amount of water entering the dryer, resulting in lower thermal energy demand for all investigated by-products. Nevertheless, the extent of the energy saving depended on the structural characteristics of the biomass, with asparagus, broccoli and artichoke exhibiting different dewatering efficiencies because of their distinct tissue organization and water distribution. The recovered liquid fractions contained appreciable amounts of water-soluble bioactive compounds, demonstrating that the proposed strategy not only can decrease the energy required for dehydration but also generate an additional value stream.

## 1. Introduction

Vegetable processing by-products are characterized by a high moisture content, which makes them highly susceptible to microbial spoilage and significantly limits their storage, transportation, and further valorization [1]. Consequently, dehydration is one of the most widely adopted stabilization strategies for converting these biomasses into shelf-stable ingredients suitable for food and non-food applications [2,3]. However, drying is also among the most energy-intensive unit operations in the food industry because a large fraction of the supplied energy is consumed to evaporate water [4]. The high latent heat of vaporization of water represents the major contribution to the overall energy demand of thermal dehydration processes. This is expected because drying requires not only sensible heat to raise the temperature of both the biomass and its water content but, more importantly, the latent heat necessary for the liquid-to-vapour phase transition of water. The latent heat of vaporization of water is less more than 2250 kJ kg^−1^ and therefore moisture evaporation constitutes the dominant energy sink during food drying [5,6].

For this reason, increasing attention has recently been devoted to process intensification strategies aimed at reducing the amount of water that must be removed by evaporation. These approaches can be broadly classified into two categories: mechanical dewatering and drying pretreatments. Mechanical dewatering encompasses physical separation processes, such as pressing, screw pressing, filtration, or centrifugation, which remove part of the water without inducing a liquid-to-vapour phase transition. Because water is physically expelled rather than evaporated, these operations require substantially less energy than thermal drying and are widely employed in wastewater treatment and biomass processing [7,8]. For instance, filter-press dewatering has been successfully applied to brewer’s spent grain, reducing its initial moisture content and improving its subsequent stabilization and handling [9,10]. Electro-kinetic dewatering coupled with mechanical pressing has been investigated for brewer’s spent grain, cauliflower trimmings, mango peel, orange peel and melon peel, demonstrating significant moisture reductions while requiring substantially lower energy than conventional thermal drying [11]. Specifically, the authors demonstrated reduction in moisture from 78% to 71% for brewer’s spent grain, from 77% to 68% for orange peel, from 80% to 73% for mango peel, from 91% to 74% for melon peel, and from 92% to 80% for cauliflower trimmings. Mechanical dewatering approaches have also been explored for fruit and vegetable processing residues, where the combination of pressure-assisted water removal and electro-osmosis increased the solids content before drying [9]. These studies collectively demonstrate that reducing the moisture load prior to evaporation is an effective strategy for decreasing the energy requirements of downstream drying processes. Most studies have mainly focused on moisture reduction as a stabilization strategy, whereas the simultaneous recovery of a liquid fraction enriched in water-soluble bioactive compounds has been scarcely explored. Although mechanical dewatering substantially reduces the energy required for moisture removal, its application is constrained by the fact that only free or weakly bound water can be efficiently removed, whereas intracellular and strongly bound water remain within the plant tissue. Consequently, thermal drying is still required to achieve the low moisture levels necessary for powder production. Moreover, the efficiency of mechanical dewatering strongly depends on the anatomical organization and physicochemical properties of the processed biomass, and the expressed liquid is generally regarded as a process stream requiring further management. The amount of water that can be mechanically removed is governed by tissue microstructure, porosity, cell-wall architecture, fiber composition, and the distribution of free and bound water within the plant matrix [12]. Consequently, by-products with different anatomical organization and physicochemical properties may exhibit markedly different dewatering performances even when processed under identical operating conditions [13]. This matrix-dependent behaviour highlights the need to evaluate mechanical dewatering strategies on a broad range of vegetable by-products differing in structural complexity before their industrial implementation. Therefore, strategies capable of simultaneously reducing the moisture load entering the dryer while valorizing the extracted liquid fraction remain largely unexplored for many vegetable processing by-products.

On the other side, drying pretreatments, including ultrasound, microwave, infrared, pulsed electric fields, and other emerging technologies, are primarily designed to modify the microstructure of plant tissues, enhance cell permeability, or improve heat and mass transfer during the subsequent drying stage [14,15,16,17]. Very recently, Jafari et al. [18] examined the drying kinetics of eggplant (*Solanum melongena* L.) peel and pulp waste in an infrared (IR)-convective dryer that operates in forced convection of unheated air (UH-air). Operating parameters, including IR power (1000, 1500, and 2000 W), sample thickness (2, 4, and 6 mm), and UH-air velocity (0.5, 1.25, and 2 m/s) and response surface methodology were evaluated to optimize drying performance. The authors verified that higher IR power, reduced sample thickness, and UH-air velocity decreased drying time while enhancing energy efficiency. In addition, greenhouse gas (GHG) emissions were minimized as IR power increased and as sample thickness and UH-air velocity decreased. Although these treatments can significantly shorten drying time and reduce overall energy consumption, they generally do not substantially decrease the amount of water that must ultimately be evaporated. Therefore, the latent heat associated with water vaporization remains the dominant contributor to the total energy demand. In this context, preliminary mechanical dewatering represents the preferred alternative because it directly lowers the moisture load entering the dryer, thereby reducing the quantity of water requiring phase change. To the best of our knowledge, the use of cold pressing as a preliminary step prior to dehydration has not yet been investigated for vegetable processing residues [19]. Besides decreasing the moisture load of the material entering the dryer, this approach offers the additional opportunity to recover a liquid fraction naturally enriched in water-soluble bioactive compounds, thereby improving both the energetic efficiency and the overall valorization potential of the biomass within a circular bioeconomy framework.

Based on this rationale, the present study aimed to evaluate the effectiveness of coupling cold-press dewatering with thermal dehydration as an innovative strategy to produce stable powders and liquid fraction from vegetable by-products while reducing the energy demand associated with the drying step. Three horticultural by-products with markedly different anatomical organization and physicochemical characteristics, asparagus, broccoli, and artichoke residues, were selected as representative case studies to assess the applicability of the proposed approach across heterogeneous plant matrices. The attention was focused on the effects of the preliminary dewatering step on the residual moisture content, on solid and liquid residue characteristics and on energy consumption.

## 2. Materials and Methods

### 2.1. Cold-Press Dewatering, Dehydration and Grinding of By-Products

Three vegetable by-products were kindly supplied by local two companies: the artichoke by ROSSOGARGANO srl, Foggia (Italy) and the asparagus and broccoli by the company FARRIS srl, Foggia (Italy). The by-products consisted of artichoke bracts and stems, broccoli stems cut into cubes, and asparagus stems cut into cubes. These materials were used as obtained from the corresponding processing operations, without additional size reduction other than the cutting described above. All the by-products were washed with tap water to remove solid residues and then immersed in chlorinated water (20 mL/L) for 5 min, rinsed with water, and allowed to air-dry. The washed by-products were stored at −18 °C. For each by-product, two independent production batches were considered. Each batch was divided into two portions: one portion was directly subjected to thermal dehydration, while the other was first subjected to cold-press dewatering and subsequently dehydrated under the same conditions. Two replicates were performed for each experimental condition. Therefore, the untreated and cold-pressed/dehydrated samples originated from the same raw-material batches, allowing the effect of cold-press dewatering to be assessed while minimizing the influence of batch-to-batch variability.

For the cold-press dewatering a low-speed screw slow juicer (Hurom H320N, Italjuicer srl, Verona, Italy) was adopted. This mechanical dewatering yielded a solid fraction, which was dehydrated and a liquid fraction (juice) that was stored under freezing conditions. Table 1 reports the cold-press process data available for each vegetable by-product, including the initial mass of material subjected to pressing, the mass of the resulting two fractions, and the duration of the cold-pressing operation. These data provide additional information on the amount of material processed, the distribution of the material between the solid and the liquid fractions, and the duration of the mechanical dewatering step.

The dehydration process of whole by-products and of the corresponding solid fraction residues was carried out for approximately 30 h in a conventional cabinet with a volume of 0.6 m^3^ (PF-SICCO80PRO, SICCOTECH, Campobasso, Italy). For dehydration the by-products were distributed over the tray as a thin and relatively uniform layer, avoiding the formation of thick accumulations of material. The drier used forced convection at atmospheric pressure, relative humidity set to 5% and temperature to 60 °C. To monitor the drying kinetics, periodically the moisture content of 5 g samples was recorded using a thermobalance (Sartorius, Göttingen, Germany) operating at 130 °C. The drying process was stopped once constant moisture values were found. Then, the dehydrated by-products were ground into fine powders (below 500 µm) using two types of laboratory grinders (KMEC Engineering, Kate Road, Anqiu City, China, and Swing type CGOLDENWALL, White Plains, NY, USA). The resulting powders were stored in plastic bags at 4 °C under vacuum conditions until further analysis.

### 2.2. Initial Moisture Content

Water content (%) was measured using a thermobalance (Sartorius, Gottingen, Germany). For each sample, 5 g were distributed uniformly on an aluminium plate and placed in a set at 130 °C.

### 2.3. Bioactive Compounds and Antioxidant Properties

As reported above, for each by-product, two independent production batches were initially considered. Each batch was divided into portions assigned to the different processing conditions. After processing, the corresponding materials from the two batches were pooled to obtain a composite sample for each sample type. Therefore, the two production batches were not treated as independent statistical replicates in the subsequent chemical analyses. The following chemicals were adopted for the nutritional quality of both solid and liquid samples: methanol anhydrous (>99.9%), Folin–Ciocalteu reagent, gallic acid monohydrate, anhydrous sodium carbonate, methanol, hydrochloric acid, 2,2-azino-bis (3-ethylbenzothiazoline-6-sulfonic acid) diammonium salt (ABTS), potassium persulfate, Trolox (6-hydroxy-2,5,7,8-tetramethylchroman-2-carboxylic acid), aluminium chloride, sodium nitrite, sodium hydroxide solution and quercetin, supplied by Sigma Aldrich (Milan, Italy). Anhydrous sodium carbonate was obtained from Carlo Erba (Milan, Italy). All reagents were of analytical grade.

To determine total phenols (TPCs), flavonoids (TFCs), and antioxidant activity (ABTS and FRAP), samples were extracted using a modified protocol based on Panza et al. [20]. For the solid fraction, TPC, TFC, ABTS and FRAP analyses were performed on the final dehydrated and ground material, corresponding to the form intended for use as a food ingredient. The liquid fractions obtained after cold-press extraction were analysed as obtained, without a preliminary drying step. While 2 g of each powder sample were mixed with 20 mL of methanol aqueous solution (80:20), the liquid samples were mixed with 80% methanol aqueous solution at 1:1 ratio. Each sample was homogenized in a 50 mL centrifuge tube, subjected to ultrasound treatment for 15 min, according to Natrella et al. [21], and centrifuged at 4 °C for 10 min at 10,000 rpm (5804R, Eppendorf, Milan, Italy) to isolate the supernatant. Then, each extract was collected, filtered (PTFE, 0.45 µm), and used for analytical determinations. All the extractions were made in triplicate (*n* = 3 analytical replicates), with appropriate dilutions.

TPC was measured using the Folin–Ciocalteu method [20] and expressed as milligrams of gallic acid equivalents per gram of dry weight (mg GAE/g dw) for solid samples and as milligrams of gallic acid equivalents per millilitre (mg GAE/mL) for liquid samples, based on a gallic acid calibration curve (3.125–100 mg/L; *R*^2^ = 0.998). TFC was assessed via the aluminium chloride method [20] and reported as milligrams of quercetin equivalents per gram of dry weight (mg QE/g dw) for solid samples and as milligrams of quercetin equivalents per millilitre (mg GAE/mL) for liquid samples, derived from a quercetin calibration curve (6.25–500 mg/L; *R*^2^ = 0.996). The ABTS assay, performed according to the methodology outlined by Re et al. [22], was expressed as milligrams of Trolox equivalents per gram of dry weight (TE/g dw) for solid samples and milligrams of Trolox equivalents per millilitre (TE/mL) for liquid samples, according to a calibration curve (12.5–500 mg/L; *R*^2^ = 0.995) using Trolox as a standard. The results from the FRAP method, as described by Marinelli et al. [23], were expressed as µmol of ferrous equivalent per gram of dry weight (µmol Fe (II)/g dw) for solid samples and µmol of ferrous equivalent per millilitre (µmol Fe (II)/mL) for liquid samples, according to calibration a curve (0.0125–1.25 mmol/L; *R*^2^ = 0.996) generated with ferrous sulfate heptahydrate as a standard. For each resulting sample, three analytical replicates were performed.

### 2.4. By-Product Water Content

The water content of the by-product was determined using the following equation:

(1) $$C\left(t\right)\;=\;\frac{W\left(t\right)-\;W_{F}}{W_{F}}\cdot 100$$

where *C(t)* is the water content of the sample at time *t*, expressed as g water per 100 g of dry matter; *W(t)* is the sample weight at time *t* (g), and *W_F_* is the final sample weight (g), obtained after drying at 130 °C until complete moisture removal.

The cumulative amount of water removed at time *t*, $M_{H_{2}O}(t)$, was calculated as

(2) $$M_{H_{2}O}\left(t\right)=C_{0}-C\left(t\right)$$

where *C*_0_ is the initial water content of the sample.

The amount of water removed at equilibrium, $M_{H_{2}O}^{\infty }$, was determined according to

(3) $$M_{H_{2}O}^{\infty }=C_{0}-C_{\infty }$$

where *C*_∞_ is the water content of the sample at the end of the dehydration process.

### 2.5. Electrical Energy Measurement for Drying, Cold-Pressing and Grinding Process

During the laboratory-scale processing of the by-products into powder, the electrical energy consumption associated with the equipment used for dehydration, cold-press dewatering and grinding was measured using an Electricity Power Meter Besvic (PMB05, Huzhou, Zhejiang Province, China). Once zeroed, the instrument measures the energy consumed from the zeroing point onward, as well as the instantaneous power. Regarding the dehydration process, the instrument was zeroed at the beginning of the process. Subsequently, at approximately 40-min intervals, the energy consumed since zeroing was recorded. By plotting the energy consumed as a function of time and fitting the data with a straight line passing through the origin, the value of γ was determined, where γ represents the slope of the fitted line and will be defined in greater detail in the following section. As reported above, the entire dehydration process was monitored for approximately 30 h. Regarding the milling and cold-press dewatering processes, the measuring instrument was zeroed at the beginning of each process, and the energy consumed was recorded at the end of the process. Thus, only a single reading was taken for each operation. It is worth noting that the same approach could not be applied to the dehydrator because, for this unit operation, the dehydration time cannot be known a priori. In fact, the calculation of the dehydration time is addressed in one of the following sections. Finally, it should be emphasized that, due to the way the energy measurements were performed, no correction for the idle or background power consumption of the different equipment used (i.e., the dehydrator, mill, and cold-press dewatering unit) was necessary.

### 2.6. Energy Consumption of the By-Product’s Dehydration Process

The procedure proposed by Le Rose et al. [24] was adopted to estimate the energy consumption per unit mass of dehydrated by-product ($\tilde{E}(t)$). Briefly, the dehydration process was described as a two-stage kinetic mechanism.

During the first stage (0 ≤ *t* ≤ *t_c_*), water removal proceeds at a constant rate according to

(4) $$M_{H_{2}O}\left(t\right)=K_{1}\cdot t$$

whereas, after the transition time (*t* > *t_c_*), the dehydration kinetics follow

(5) $$M_{H_{2}O}\left(t\right)=K_{1}\cdot t_{c}+K_{2}\cdot \left\{1-\exp\left[-\left(t-t_{c}\right)\cdot \left(\frac{K_{1}}{K_{2}}\right)\right]\right\}$$

where *K*_1_ is the water desorption rate during the first dehydration stage ($\frac{g\;\mathrm{desorbed}\;\mathrm{water}}{100\;g\;\mathrm{dry}\;\mathrm{matter}}\cdot \frac{1}{\min}$), *K*_2_ is the maximum amount of water removed during the second stage ($\frac{g\;\mathrm{desorbed}\;\mathrm{water}}{100\;g\;\mathrm{dry}\;\mathrm{matter}}$), *t_c_* is the transition time between the two dehydration stages, and *t* is the dehydration time. A detailed derivation of the model together with the underlying assumptions is provided by Conte et al. [25].

As previously demonstrated by Panza et al. [26], the dehydration time required to achieve a prescribed dehydration extent (*t*_ext%_) can be expressed as a function of the dehydration extent (ext%). For $0\;\leq \;\mathrm{ext}\%\;\leq \;\frac{K_{1}\cdot t_{c}}{M_{H_{2}O}^{\infty }}\cdot 100$ the following relationship applies:

(6) $$t_{\mathrm{ext}\%}\;=\;\frac{M_{H_{2}O}^{\infty }}{K_{1}\cdot 100}\cdot \mathrm{ext}\%$$

whereas for larger dehydration extents, $\mathrm{ext}\%\;>\;\frac{K_{1}\cdot t_{c}}{M_{H_{2}O}^{\infty }}\cdot 100$, the dehydration time is given by

(7) $$t_{\mathrm{ext}\%}=t_{c}-\ln\left(\frac{K_{1}\cdot t_{c}+K_{2}-\mathrm{ext}\%\cdot \frac{M_{H_{2}O}^{\infty }}{100}}{K_{2}}\right)\cdot \frac{K_{2}}{K_{1}}$$

Based on these expressions, the specific energy consumption ($i.e.,\;\tilde{E}(t)$) can be directly related to the dehydration extent. For dehydration extents below the transition point, $0\;\leq \;\mathrm{ext}\%\;\leq \;\frac{K_{1}\cdot t_{c}}{M_{H_{2}O}^{\infty }}\cdot 100$, the specific energy consumption is calculated as

(8) $$\tilde{E}\left(\mathrm{ext}\%\right)=\frac{\gamma \cdot \left(\frac{M_{H_{2}O}^{\infty }}{K_{1}\cdot 100}\cdot \mathrm{ext}\%\right)}{m_{\mathrm{Tot}}^{0}\cdot \left(1-\frac{x_{\mathrm{dm}}^{0}}{100}\cdot \mathrm{ext}\%\cdot \frac{M_{H_{2}O}^{\infty }}{100}\right)}$$

whereas for higher dehydration extents, $\mathrm{ext}\%\;>\;\frac{K_{1}\cdot t_{c}}{M_{H_{2}O}^{\infty }}\cdot 100$, the corresponding expression becomes

(9) $$\tilde{E}\left(\mathrm{ext}\%\right)=\frac{\gamma \cdot \left[t_{c}-\ln\left(\frac{K_{1}\cdot t_{c}+K_{2}-\mathrm{ext}\%\cdot \frac{M_{H_{2}O}^{\infty }}{100}}{K_{2}}\right)\cdot \frac{K_{2}}{K_{1}}\right]}{m_{\mathrm{Tot}}^{0}\cdot \left(1-\frac{x_{\mathrm{dm}}^{0}}{100}\cdot \mathrm{ext}\%\cdot \frac{M_{H_{2}O}^{\infty }}{100}\right)}$$

where *γ* is the power supplied to the dehydrator, $m_{\mathrm{Tot}}^{0}$ is the initial mass of the by-product, and $x_{\mathrm{dm}}^{0}$ is the initial dry matter mass fraction. A complete derivation of Equations (8) and (9), together with the procedure used to estimate the specific energy consumption, can be found in the work of Le Rose et al. [24].

### 2.7. Statistical Analysis

Experimental data from chemical analyses and fitting parameters were compared by a one-way analysis of variance (HSD of Tukey), with the option of homogeneous groups (*p* < 0.05), to determine significant differences among samples. The statistical analysis for TPC, TFC, ABTS and FRAP was performed on the three analytical replicates (*n* = 3) for each sample type, comparing, within each by-product, the two resulting solid matrices (dried by-product powder and cold-pressed solid residue subsequently subjected to drying) and the liquid fraction obtained after cold pressing. The statistical analysis was therefore not intended to estimate independent main effects of vegetable type and processing strategy or their interaction. JMP 18 for Windows (JMP Statistical Discovery LLC 920 SAS Campus Drive, Cary, NC 27513, USA) was used.

## 3. Results and Discussion

### 3.1. Bioactive Compounds and Antioxidant Properties of Solid and Liquid Fractions

The bioactive profile differed significantly among the solid samples reported in Table 2, with clear differences according to the vegetable by-product and processing condition. Among the samples directly subjected to dehydration, asparagus showed the highest TPC, significantly higher than all other samples (*p* < 0.05). Broccoli and artichoke showed similar TPC values (approximately 5.7 mg GAE/g dw), whereas the cold-pressed residues of broccoli, asparagus and artichoke showed lower values. For each by-product, the cold-pressed residue showed a significantly lower TPC than the corresponding directly dehydrated sample (*p* < 0.05). The high TPC observed in asparagus is consistent with the recognized presence of phenolic compounds in asparagus and its processing residues [27,28], while the results for artichoke are consistent with its established potential as a source of phenolic and nutraceutical compounds [29,30].

TFC showed a similar trend, although the differences among matrices were more pronounced. Asparagus without cold pressing showed the highest value, followed by artichoke without cold pressing, whereas broccoli showed the lowest values before and after cold pressing. According to the statistical grouping, the two broccoli samples were not significantly different (*p* > 0.05), whereas all other sample types differed significantly from each other (*p* < 0.05). The reduction observed in the cold-pressed residues is compatible with the removal of water-soluble compounds during mechanical dewatering. However, in the absence of compound-specific analyses and a complete mass balance, this decrease cannot be interpreted quantitatively as the transfer or recovery of flavonoids in the liquid fraction.

The antioxidant assays broadly followed the trends observed for TPC and TFC. Asparagus without cold pressing showed the highest ABTS and FRAP values, while broccoli generally showed the lowest antioxidant activity. For both ABTS and FRAP, the cold-pressed residues exhibited significantly lower values (*p* < 0.05) than the corresponding directly dehydrated samples, although the magnitude of the reduction differed among matrices. This matrix-dependent response is consistent with the known differences in the antioxidant characteristics of asparagus and broccoli [27] and with the influence that drying conditions can exert on bioactive compounds and antioxidant activity in broccoli [31]. To sum up, cold pressing was associated with a decrease in the concentration of phenolic and flavonoid compounds and antioxidant activity retained in the solid residue. Nevertheless, all cold-pressed solids retained measurable bioactive compounds and antioxidant activity, indicating that the mechanical treatment did not eliminate their functional potential. From an upcycling perspective, the findings of the current study are relevant because the proposed process generates complementary solid and liquid streams. The solid fraction obtained after dehydration and grinding should therefore be regarded primarily as a fibre-rich vegetable ingredient rather than as a concentrated extract of bioactive compounds. Its potential value lies in the combined presence of dietary fibre and residual phenolic compounds and antioxidant activity, together with the possibility of incorporating dehydrated vegetable processing biomass into food formulations. The liquid fraction represents another potentially valuable source of soluble constituents. Potential applications include pasta, as well as bakery and other cereal-based products, although specific technological and sensory studies are required to establish suitable food matrices and incorporation levels. Such an integrated approach is consistent with current strategies for converting food-processing by-products into multiple higher-value streams [32]. Further mass-balance and compound-specific analyses would be required to establish the fate of individual bioactive compounds between the two fractions. When considered together, the results obtained for the solid and liquid fractions indicate that the mechanical dewatering step generated two streams with different levels of phenolic and flavonoid equivalents and antioxidant activity. However, these concentration data cannot be used to establish the recovery or partitioning of individual bioactive compounds between the two streams.

With regard to liquid fraction, although mechanical dewatering may transfer water-soluble constituents from the original by-product to the liquid stream, the present study did not quantify sugars, minerals, vitamins or other nutrients; therefore, no conclusion can be drawn regarding the extent or nutritional relevance of such partitioning. TPC, TFC and antioxidant activity analyses highlight that the liquid fractions also showed marked differences in bioactive profiles depending on the vegetable by-product (Table 3). Artichoke exhibited the highest values for all the investigated parameters. Broccoli showed the lowest TPC, TFC and ABTS activity, whereas asparagus showed intermediate values. For FRAP, broccoli and asparagus did not differ significantly (*p* > 0.05). The particularly high bioactive content observed in the artichoke liquid fraction is consistent with the recognized richness of artichoke tissues in phenolic and flavonoid compounds and with previous evidence showing the occurrence of these compounds in artichoke juice and other processing fractions [29,30]. During cold pressing, these compounds are readily released from disrupted cells and preferentially partition into the aqueous phase, leading to an enrichment of the juice and a concomitant depletion of the solid residue. The higher TPC and TFC of the liquid fraction therefore reflects the greater extractability and water affinity of artichoke phenolics compared with those of broccoli and asparagus, where a larger proportion of phenolics remain associated with cell wall polysaccharides and other insoluble structural components.

When considered together with the results obtained for the solid fractions, the data indicate that the proposed cold-pressing step generates two streams with distinct characteristics. Although the cold-pressed solid residues generally showed lower concentrations of phenolic and flavonoid compounds and antioxidant activity than the corresponding directly dehydrated materials, the resulting liquid fractions contained measurable amounts of these bioactive constituents. These findings support the potential of the proposed integrated approach to generate complementary valorization streams from the same by-product, with the solid residue representing a fibre-rich material and the liquid fraction providing a source of soluble bioactive compounds. While previous studies have characterized the bioactive potential of different solid or liquid fractions of plant materials [29,30], the simultaneous generation and valorization of the two streams through mechanical dewatering followed by dehydration, as proposed here, has received limited attention. Therefore, the approach proposed in this study is consistent with the increasing interest in upcycling food-processing by-products into multiple higher-value products.

### 3.2. Specific Energy Consumption for By-Product Powder Production

As outlined in the Materials and Methods section, the production of by-product powders involves three unit-operations that contribute to the total specific energy consumption: cold-press dewatering (when performed), dehydration, and grinding. While the energy consumption associated with cold-press dewatering and grinding was determined experimentally, the energy required for dehydration was estimated from the dehydration kinetics using the mathematical model previously described.

**Figure 1:**
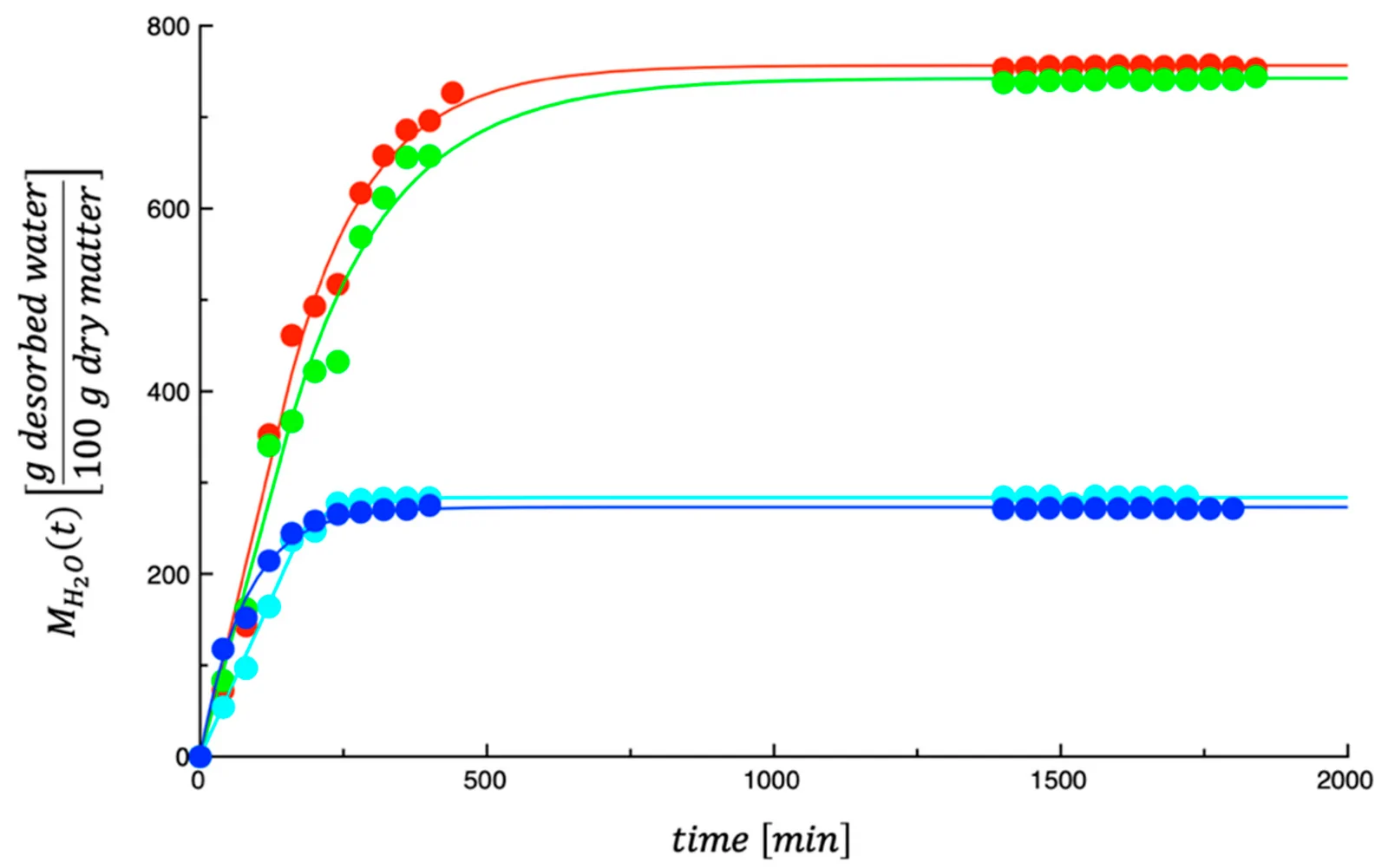
Broccoli by-products dehydration kinetics: ● first test, ● second test. Broccoli solid residue dehydration kinetics: ● first test 1; ● second test. Curve to fit broccoli by-products dehydration kinetics: ▬ first test, ▬ second test. Curve to fit broccoli solid residue dehydration kinetics: ▬ first test, ▬ second test.

**Figure 2:**
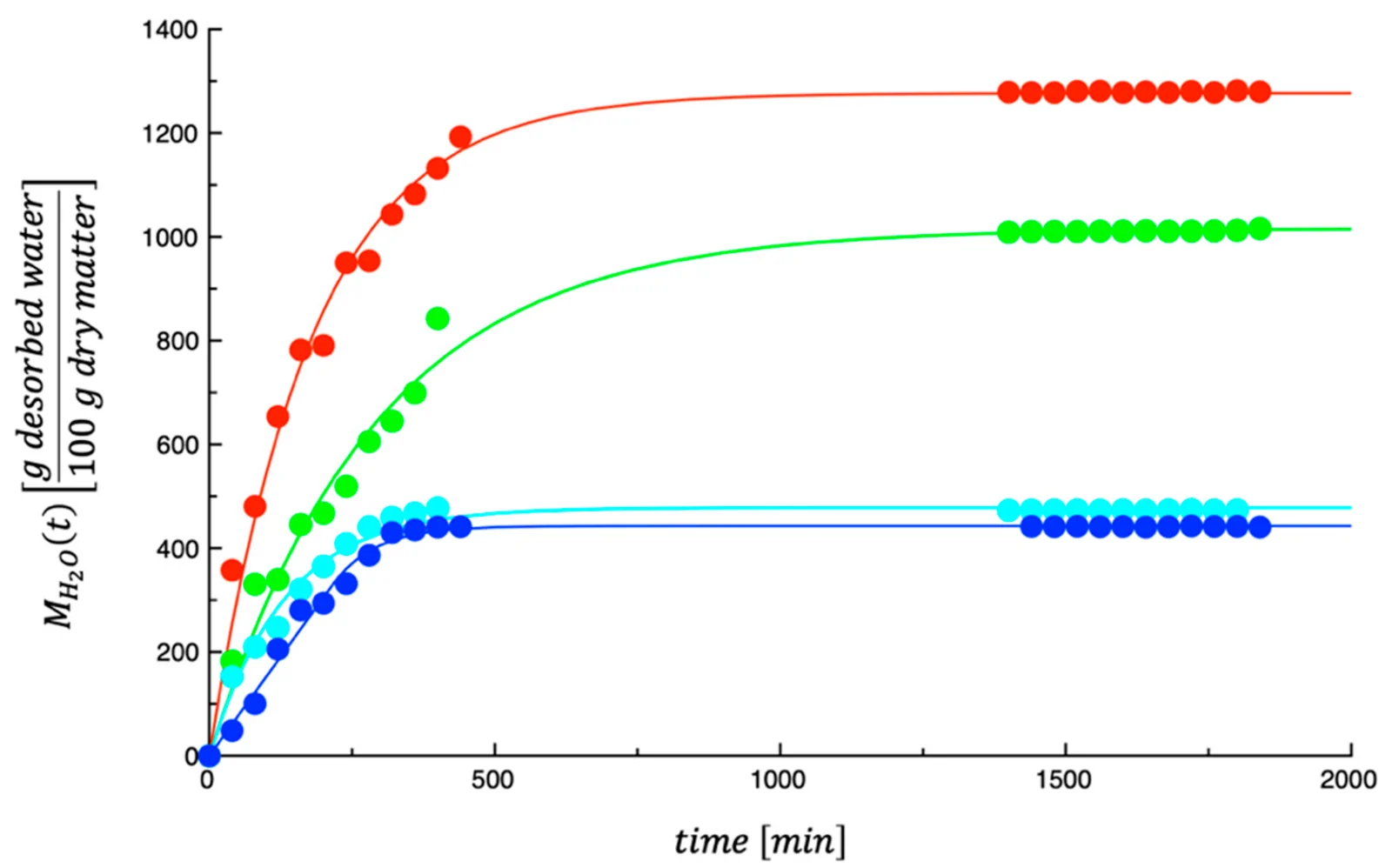
Asparagus by-products dehydration kinetics: ● first test, ● second test. Asparagus solid residue dehydration kinetics: ● first test 1; ● second test. Curve to fit asparagus by-products dehydration kinetics: ▬ first test, ▬ second test. Curve to fit asparagus solid residue dehydration kinetics: ▬ first test, ▬ second test.

**Figure 3:**
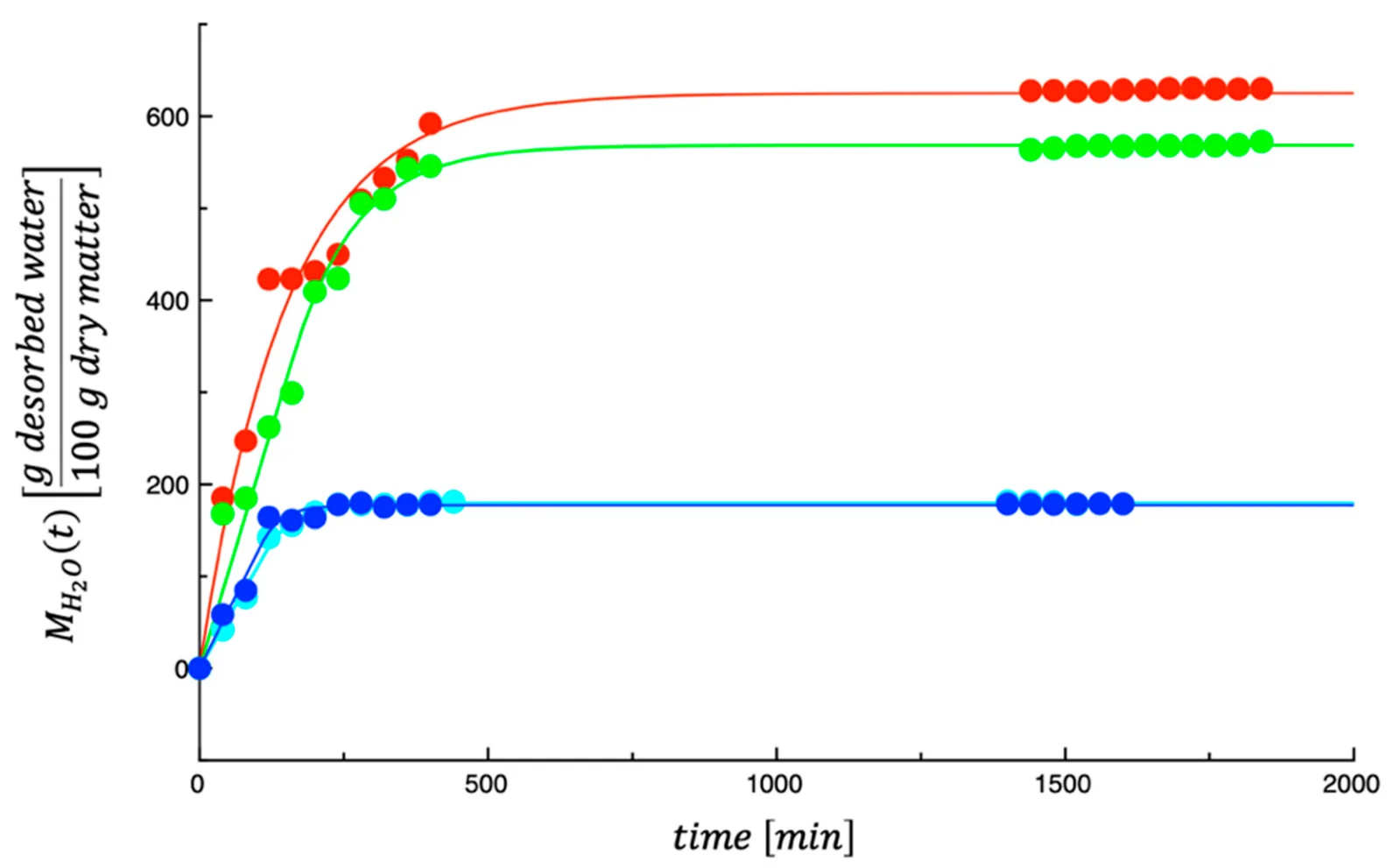
Artichoke by-products dehydration kinetics: ● first test, ● second test. Artichoke solid residue dehydration kinetics: ● first test 1; ● second test. Curve to fit artichoke by-products dehydration kinetics: ▬ first test, ▬ second test. Curve to fit artichoke solid residue dehydration kinetics: ▬ first test, ▬ second test.

Figure 1, Figure 2 and Figure 3 compare the water desorption kinetics of broccoli, asparagus, and artichoke by-products with and without the mechanical dewatering process. For each sample type, the results obtained from both experimental trials are reported. As expected, the cold-press dewatering process substantially affected the dehydration behaviour of the by-products. In addition, the three by-products exhibited distinct equilibrium water desorption capacities. Their moisture content differed significantly among the investigated matrices, with asparagus showing the highest amount of water removed at equilibrium and artichoke the lowest. This trend is consistent with previous findings reported in the literature, indicating that the observed differences are not unexpected. However, it should be emphasized that these comparisons are only applicable to the whole dehydrated by-products. Although several studies have investigated mechanical dewatering technologies to reduce the moisture content of food by-products before thermal processing [1], the use of cold pressing for this specific purpose appears to be novel, thus not making possible direct comparisons with the extracted samples.

With regard to thermal dehydration of the whole by-products, an earlier study conducted by the same research group [27] reported the same ranking for the corresponding dehydrated raw by-products, confirming the reproducibility of this experimental evidence across different sample sets. Furthermore, the study by Ayub et al. [33], which investigated dehydrated artichoke and olive by-products, reported moisture values for artichoke residues very comparable to those obtained in the current work for whole artichoke by-products. The persistence of these differences after dehydration is likely attributable to the intrinsic structural and compositional characteristics of the plant tissues rather than by variability in the dehydration process itself. Asparagus tissues are characterized by a more porous structure and a higher proportion of parenchymatous tissue, which can retain water more strongly and reduce drying efficiency [34]. These structural features govern moisture transport during dehydration and may lead to different drying behaviour compared with tissues having a denser and more lignified architecture. Indeed, the influence of tissue microstructure, porosity and cell-wall organization on effective moisture diffusivity and drying kinetics has been widely recognized in plant materials [35]. The lower residual moisture observed in artichoke by-products may be associated with their distinctive lignocellulosic composition. Artichoke processing residues are known to contain high amounts of structural polysaccharides and lignin, which determine their mechanical properties and tissue architecture [29,36,37]. Since moisture transport during dehydration is strongly governed by cell-wall composition and microstructure, these anatomical differences likely contributed to the lower equilibrium moisture content observed after drying [38]. Broccoli by-products exhibited an intermediate residual moisture content between asparagus and artichoke. This evidence may be explained by the anatomical and compositional characteristics of broccoli stems. Unlike asparagus, whose tissues are predominantly parenchymatous, broccoli stems contain both parenchymatous tissues and well-developed vascular bundles, together with appreciable amounts of structural polysaccharides, including cellulose, hemicellulose, pectins, and lower levels of lignin than typically reported for artichoke by-products. Consequently, broccoli exhibits an intermediate tissue organization that likely results in moisture transport properties between those of asparagus and artichoke. This interpretation is consistent with studies describing the cell-wall composition of broccoli stems and the role of tissue architecture in determining their physicochemical and technological properties [39,40,41].

Regarding the repeatability of the experiments, some differences between the two dehydration trials were observed for all the investigated by-products. Although the drying conditions were kept constant, slight differences in the desorption kinetics were observed among different batches of the same by-product. In most cases, these differences were relatively small. However, a more pronounced variability was found for asparagus, where the two dehydration curves differed more substantially. It should be emphasized that the two trials for each by-product were performed using different production batches, without any selection or standardization of raw material. Consequently, the observed variability likely reflects the natural heterogeneity of the by-products rather than experimental uncertainty. This is particularly evident for asparagus and makes the proposed approach more representative of real industrial conditions, where batch-to-batch variability is unavoidable. Such behaviour is expected for plant-derived materials, whose physicochemical properties are inherently affected by biological variability. Differences in cultivar, maturity stage, seasonal and environmental growing conditions, and post-harvest handling may alter tissue microstructure, initial moisture content, porosity, and cell-wall composition, thereby affecting effective moisture diffusivity and moisture transport during dehydration. Similar considerations have been highlighted in previous reviews on food drying, which identify the intrinsic variability of plant materials as one of the major sources of variation in drying kinetics and effective moisture diffusivity [42,43,44,45].

The continuous lines correspond to the best fits of Equations (4) and (5) to the experimental data, while the estimated model parameters are summarized in the Supplementary Materials (Tables S1–S3). The mean relative deviation modulus $\left(\bar{E}\%\right)$ was used to measure the goodness of fit [46]:

(10) $$\bar{E}\%=\frac{100}{N}\cdot \sum_{i=1}^{i=N}\frac{\left|M_{i}^{\exp}-\;M_{i}^{\mathrm{pred}}\right|}{M_{i}^{\exp}}$$

where *N* is the number of experimental data. $M_{i}^{\exp}$ is the experimental value. $M_{i}^{\mathrm{pred}}$ is the predicted value. The calculated $\bar{E}\%$ values are reported in the Supplementary Materials (Tables S1–S3). They ranged from 1.95 to 5.74, indicating a satisfactory goodness of fit over all the experimental conditions investigated. These results demonstrate that the proposed model accurately describes the dehydration kinetics of the three by-products.

The specific energy consumption of the individual processing stages (cold-press dewatering, dehydration, and grinding) and the corresponding total value (*E_Tot_*) are reported in Table 4. For consistency, all energy values are expressed per gram of powder obtained after grinding. The specific dehydration energy (*E_D_*) was calculated from $\tilde{E}(99\%)$ and converted to the final powder basis, thereby accounting for the mass changes occurring during the subsequent processing steps. The results clearly show that the energy demand varies considerably among the three unit-operations. In all cases, dehydration represents the dominant energy-consuming step, owing to the large amount of energy required to remove water through liquid-to-vapor phase transition. This is expected because drying requires not only sensible heat to raise the temperature of both the biomass and its water content but, more importantly, the latent heat necessary for the liquid-to-vapour phase transition of water. Since the latent heat of vaporization of water is approximately 2257–2260 kJ kg^−1^, moisture evaporation constitutes the dominant energy sink during food drying, making dehydration one of the most energy-intensive unit operations in food processing [5,47]. The energy consumption associated with the remaining processing steps is at least one order of magnitude lower than that required for dehydration.

As shown in Table 4, the inclusion of the cold-press dewatering step significantly reduced the total specific energy consumption for all the three by-products investigated. The observed reductions were substantial and statistically significant in every case (*p* < 0.05). It should be emphasized that the statistical analysis reported in the table was carried out exclusively to assess the effect of including or omitting the dewatering step on the specific energy consumption of powder production. This finding is consistent with expectations, as the total specific energy consumption is primarily determined by the energy required for dehydration. The cold-press dewatering step substantially lowers the initial water content of the by-product entering the dehydrator, thereby reducing the amount of water that must be evaporated. Thus, the energy demand for the dehydration stage, and, therefore, the overall specific energy consumption, is significantly reduced.

**Figure 4:**
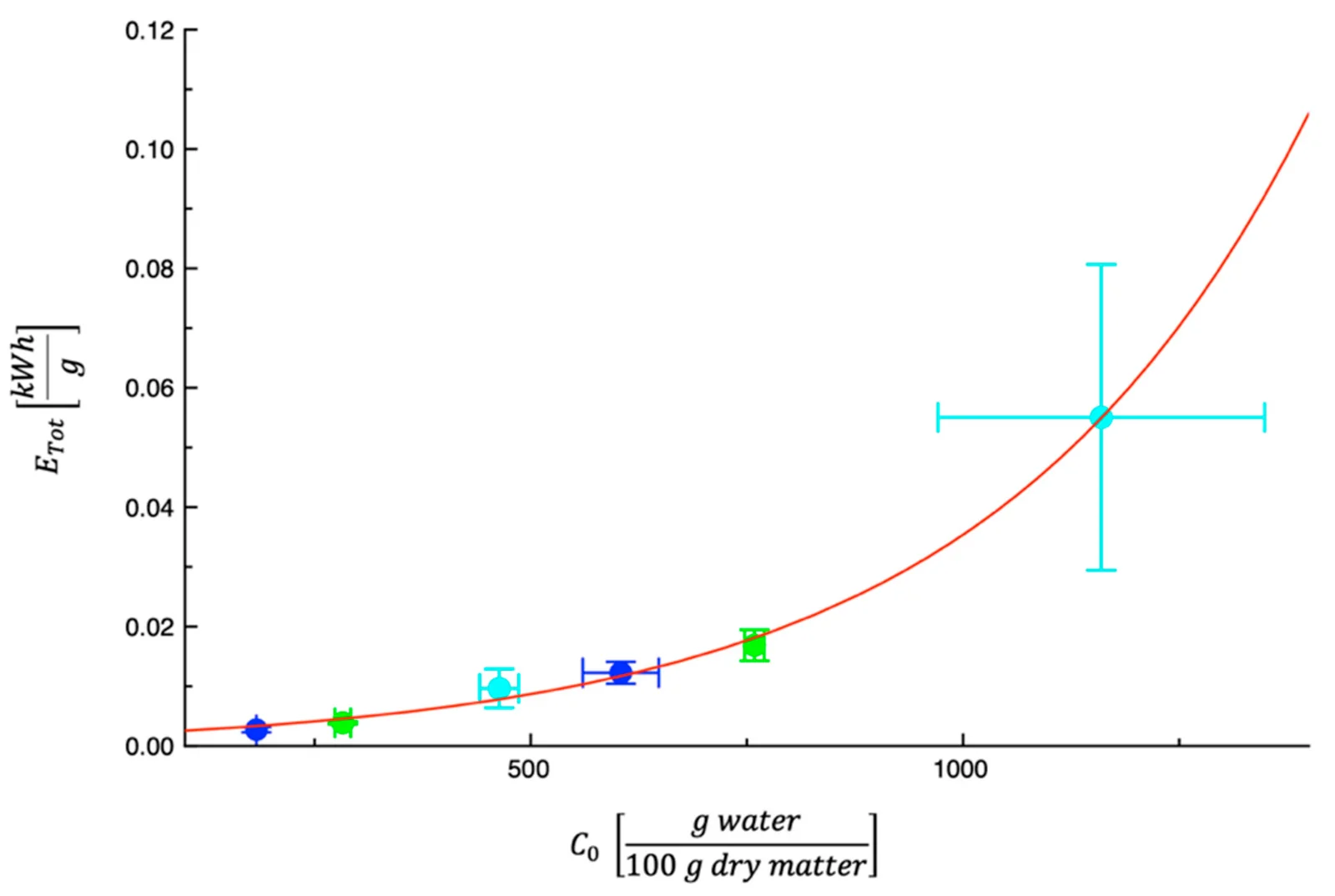
Total specific energy consumption (*E_Tot_*) as function of the initial water content of each by-product entering the dehydrator (*C*_0_) for both tests. ● Broccoli by-products, ● asparagus by-products, ● artichoke by-products. The curve shown in the figure is intended only as a visual guide to the eye and does not represent the fit of any mathematical model to the experimental data.

The above findings are further corroborated by Figure 4, which illustrates the dependence of the total specific energy consumption (*E_Tot_*) on the initial water content of the by-product entering the dehydrator (*C*_0_). Figure 4 includes both horizontal and vertical error bars, since both the independent and dependent variables are expressed as mean ± standard deviation, as described in the Materials and Methods section. As previously discussed, the untreated asparagus sample exhibited noticeably larger error bars than the other samples. This behaviour is most likely associated with the inherent batch-to-batch variability of the raw material, since the two experimental trials were performed using different production batches without prior standardization [38]. As shown in Figure 4, the relationship between *E*_tot_ and the initial water content is nonlinear, with the total specific energy consumption increasing at a rate greater than linear. Although this trend may appear inconsistent with the previous observations, the apparent discrepancy arises from the fact that *E*_tot_ is expressed as a specific energy consumption. Consequently, it is influenced not only by the total energy consumed during processing, but also by the mass of powder recovered at the outlet of the production process.

**Figure 5:**
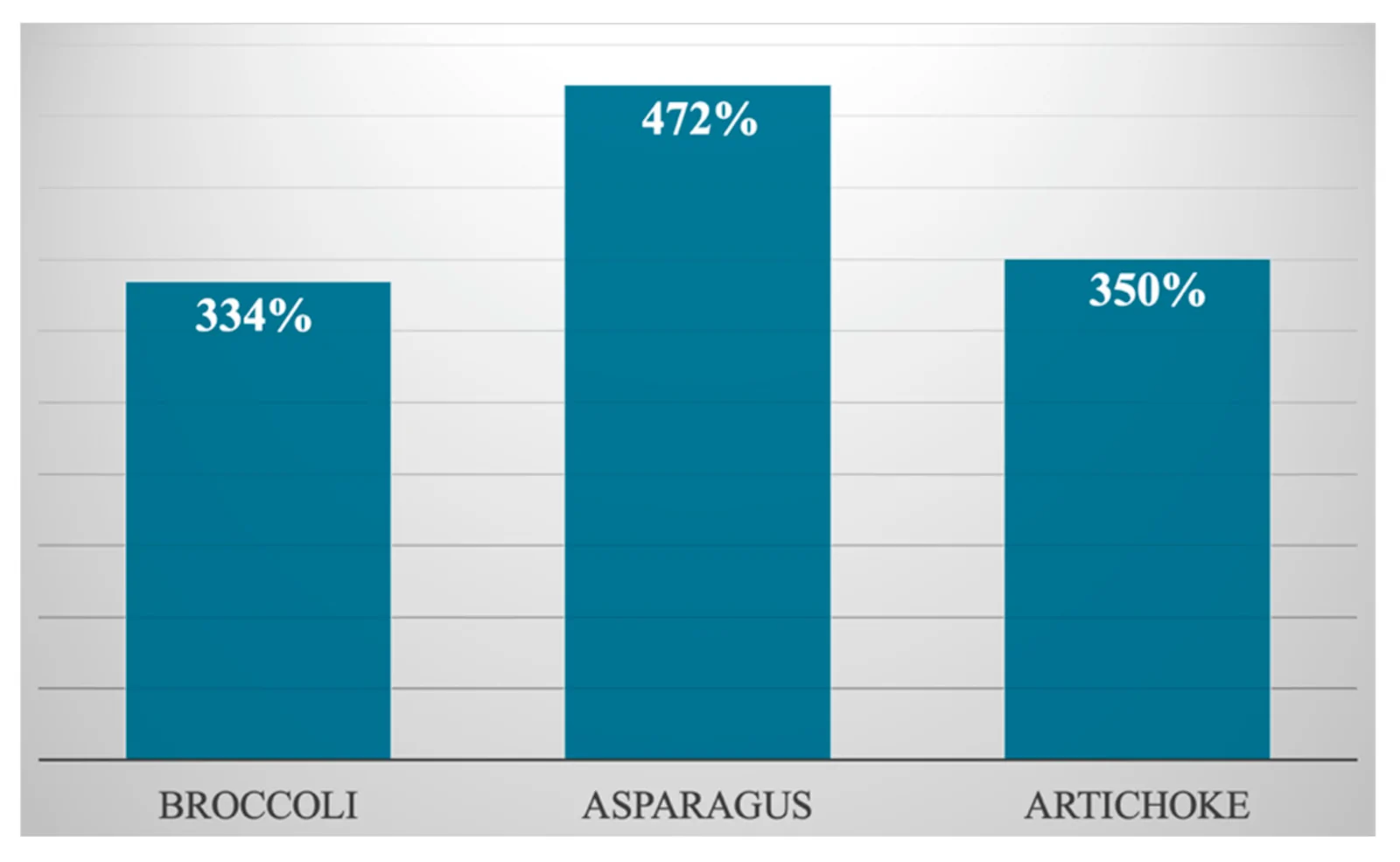
The additional energy consumption (Δ%) associated with omitting the cold-press dewatering step from the by-product powder production process.

To quantify the additional energy consumption associated with omitting the cold-press dewatering step from the by-product powder production process, the following variable was defined:

(11) $$\Delta \%=\frac{E_{\mathrm{Tot}}^{\mathrm{no}-\mathrm{extract}}\;-\;E_{\mathrm{Tot}}^{\mathrm{extract}}}{E_{\mathrm{Tot}}^{\mathrm{extract}}}\cdot 100$$

where Δ% denotes the percentage increase in specific energy consumption associated with the omission of the cold-press dewatering step, $E_{\mathrm{Tot}}^{\mathrm{no}-\mathrm{extract}}$ and $E_{\mathrm{Tot}}^{\mathrm{extract}}$ are the specific energy consumptions required for powder production without and with the dewatering step, respectively. The specific energy consumption values reported in Table 4 were used to estimate Δ% for the three investigated by-products. The resulting values are presented in Figure 5, illustrating the additional energy consumption associated with omitting the cold-press dewatering step from the by-product powder production process. Data reported in Figure 5 clearly show that the absence of the cold-press dewatering step would increase the specific energy consumption by more than 300% for all the investigated by-products, reaching values above 400% for asparagus. These results suggest that cold-press dewatering can substantially reduce the energy requirements associated with the subsequent dehydration of the investigated by-products, supporting its potential as an energy-saving pretreatment.

## 4. Conclusions

This study demonstrated that integrating cold-press dewatering with thermal dehydration can represent an effective strategy for by-product valorization. The proposed approach enabled the simultaneous production of powder and liquid fractions containing valuable water-soluble bioactive compounds. The results highlighted that the response to the integrated process was strongly influenced by the intrinsic characteristics of the processed biomass. Asparagus, broccoli and artichoke by-products exhibited different dewatering efficiencies and drying behaviours, reflecting their distinct anatomical organization and physicochemical composition. Despite these matrix-dependent differences, the proposed process proved effective for all the investigated by-products, confirming its applicability to heterogeneous vegetable residues. From a compositional perspective, the solid residues obtained after cold-press dewatering retained nutritional value. At the same time, the extracted liquid fraction from artichoke demonstrated that mechanical dewatering could be an opportunity to recover high-value ingredients. The energy analysis clearly identified dehydration as the most energy-demanding operation of the entire process because of the energy required for water evaporation. The inclusion of the cold-press dewatering step significantly reduced the total specific energy consumption for all three investigated by-products by decreasing the amount of water entering the drying stage. Moreover, the relationship between total specific energy consumption and the initial moisture content was found to be nonlinear, with energy demand increasing at a rate greater than proportional to water content. Consequently, omitting the cold-press dewatering step would increase the specific energy consumption by more than 300% for all investigated by-products, exceeding 400% in the case of asparagus. These findings provide compelling evidence that preliminary mechanical dewatering could be a key enabling technology for the energy-efficient recycling of vegetable by-product, even though a comprehensive techno-economic and life cycle assessments, together with pilot-scale validation, would provide valuable information for evaluating the industrial feasibility and the concrete environmental benefits of this integrated mechanical dewatering–dehydration strategy.

Future research should also investigate the applicability of this approach to a wider range of fruit and vegetable by-products characterized by different anatomical structures and moisture distributions. Further studies should also focus on optimizing cold-press operating conditions to maximize both water removal and bioactive compound recovery, as well as on integrating the recovered liquid fraction into the formulation of functional foods, beverages or natural ingredients.

## Figures and Tables

**Table 1:** Initial by-product mass, amount of solid fraction, liquid fraction and pressing time for the cold-press dewatering applied to broccoli, artichokes and asparagus by-products.

	Initial By-Product Mass [g]	Solid Fraction [g]	Liquid Fraction [g]	Cold-Pressing Time [min]
Broccoli	35,130.31 ± 3268.6	6760.24 ± 518.32	27,354.28 ± 2114.92	170 ± 2.6
Artichokes	23,353.99 ± 596.6	6259.65 ± 5.85	16,397.37 ± 672.73	331 ± 9.9
Asparagus	23,413.40 ± 378.9	6414.39 ± 69.02	16,028.76 ± 1074.02	132.5 ± 14.9

Data are reported as mean ± standard deviation of two replicated cold-press dewatering processes.

**Table 2:** Total Phenol Content (TPC), Total Flavonoid Content (TFC) and Antioxidant activity (ABTS and FRAP assay) of powder obtained from direct drying process (no cold-press) and with cold-press dewatering pretreatment (cold-press), from broccoli, asparagus and artichoke by-products.

Sample		TPC $\left[\frac{\mathrm{mg GAE}}{\mathrm{g dw}}\right]$	TFC $\left[\frac{\mathrm{mg QE}}{\mathrm{g dw}}\right]$	ABTS $\left[\frac{\mathrm{mg Trolox}}{\mathrm{g dw}}\right]$	FRAP $\left[\frac{\mu \mathrm{mol Fe}(\mathrm{II})}{\mathrm{g dw}}\right]$
Broccoli	no cold-press	5.76 ± 0.30 ^b^	1.40 ± 0.13 ^e^	13.82 ± 0.36 ^b^	32.98 ± 0.94 ^c^
	cold-press	1.84 ± 0.10 ^c^	0.79 ± 0.06 ^e^	6.81 ± 0.58 ^d^	6.66 ± 0.57 ^e^
Asparagus	no cold-press	13.1 ± 0.71 ^a^	5.54 ± 0.4 ^a^	31.86 ± 1.79 ^a^	61.56 ± 2.08 ^a^
	cold-press	5.68 ± 0.41 ^b^	2.71 ± 0.2 ^d^	14.76 ± 0.82 ^b^	23.5 ± 1.03 ^d^
Artichoke	no cold-press	5.71 ± 0.40 ^b^	4.6 ± 0.13 ^b^	10.54 ± 0.57 ^c^	56.86 ± 0.59 ^b^
	cold-press	2.55 ± 0.12 ^c^	3.52 ± 0.26 ^c^	5.90 ± 0.69 ^d^	20.45 ± 1.90 ^d^

Data are reported as means ± standard deviation. Data in each column with different superscript letters are statistically different (*p* < 0.05).

**Table 3:** Total Phenol Content (TPC), Total Flavonoid Content (TFC) and Antioxidant activity (ABTS and FRAP assay) of liquid residue obtained from cold-press dewatering pretreatment applied to broccoli, asparagus and artichoke by-products.

Sample		TPC $\left[\frac{\mathrm{mg GAE}}{\mathrm{mL}}\right]$	TFC $\left[\frac{\mathrm{mg QE}}{\mathrm{mL}}\right]$	ABTS $\left[\frac{\mathrm{mg Trolox}}{\mathrm{mL}}\right]$	FRAP $\left[\frac{\mu \mathrm{mol Fe}(\mathrm{II})}{\mathrm{mL}}\right]$
Broccoli	Liquid residue	0.33 ± 0.03 ^c^	0.07 ± 0.01 ^c^	0.95 ± 0.15 ^c^	1.75 ± 0.29 ^b^
Asparagus	Liquid residue	0.67± 0.14 ^b^	0.31 ± 0.01 ^b^	1.39 ± 0.07 ^b^	2.32 ± 0.24 ^b^
Artichoke	Liquid residue	1.40 ± 0.15 ^a^	1.59 ± 0.15 ^a^	2.43 ± 0.12 ^a^	9.29 ± 0.34 ^a^

Data are reported as means ± standard deviation. Data in each column with different superscript letters are statistically different (*p* < 0.05).

**Table 4:** *E_D_* is the energy consumed by the dehydrator per gram of product exiting the grinder; *E_E_* is the energy consumed by the cold-press dewatering per gram of product exiting the grinder; *E_G_* is the energy required for grinding per gram of product exiting the grinder, *E_Tot_* is the total energy consumed per gram of product exiting the grinder (i.e., *E_Tot_* = *E_D_* + *E_E_* + *E_G_*).

	Broccoli By-Products	Broccoli Solid Residue	Asparagus By-Products	Asparagus Solid Residue	Artichoke By-Products	Artichoke Solid Residue
*E_D_* $\left[\frac{\mathrm{kWh}}{g}\right]$	1.7 × 10^−2^ ± 2.6 × 10^−3 a^	3.5 × 10^−3^ ± 2.3 × 10^−4 b^	5.5 × 10^−2^ ± 2.6 × 10^−2 A^	9.3 × 10^−3^ ± 3.3 × 10^−3 B^	1.2 × 10^−2^ ± 1.9 × 10^−3 ɑ^	2.1 × 10^−3^ ± 4.4 × 10^−4 ß^
*E_E_* $\left[\frac{\mathrm{kWh}}{g}\right]$	-	2.8 × 10^−4^ ± 1.2 × 10^−5^	-	2.1 × 10^−4^ ± 1.7 × 10^−5^	-	4.1 × 10^−4^ ± 3.8 × 10^−5^
*E_G_* $\left[\frac{\mathrm{kWh}}{g}\right]$	5.5 × 10^−5^ ± 4.3 × 10^−6 b^	9.4 × 10^−5^ ± 4.3 × 10^−6 a^	6.1 × 10^−5^ ± 2.5 × 10^−6 B^	1.1 × 10^−4^ ± 1 × 10^−5 A^	1.3 × 10^−4^ ± 4.5 × 10^−5 ß^	2.9 × 10^−4^ ± 1.9 × 10^−5 ɑ^
*E_Tot_* $\left[\frac{\mathrm{kWh}}{g}\right]$	1.7 × 10^−2^ ± 2.6 × 10^−3 a^	3.9 × 10^−3^ ± 2.3 × 10^−4 b^	5.5 × 10^−2^ ± 2.6 × 10^−2 A^	9.7 × 10^−3^ ± 3.3 × 10^−3 B^	1.3 × 10^−2^ ± 1.8 × 10^−3 ɑ^	2.7 × 10^−3^ ± 4.2 × 10^−4 ß^

Data are presented as mean ± standard deviation (*n* = 2) for each by-product. In each row, different lowercase letters denote statistically significant differences (*p* < 0.05) between the two broccoli samples, different uppercase letters between the two asparagus samples, and different lowercase Greek letters between the two artichoke samples.

## Data Availability

The original contributions presented in the study are included in the article/Supplementary Materials, further inquiries can be directed to the corresponding author.

## Supplementary Materials

The following supporting information can be downloaded at: https://www.mdpi.com/article/10.3390/foods15193481/s1, Table S1: Model parameters of Equations (4) and (5) fitted to the experimental data of broccoli by-products; Table S2: Model parameters of Equations (4) and (5) fitted to the experimental data of asparagus by-products; Table S3: Model parameters of Equations (4) and (5) fitted to the experimental data of artichoke by-products.
