# Supplementary material for: Cold-Pressing as an Energy-Saving Dewatering Pretreatment for the Valorization of Vegetable Processing By-Products
Source: Foods. 2026 Sep 29;15(19):3481. doi: 10.3390/foods15193481 (PMC13665568; doi:10.3390/foods15193481)
Supplement: Supplementary file 1 [file foods-15-03481-s001.zip › foods-4575453-supplementary.pdf]

foods

MDPI

Supplementary material

Table S1: Model parameters of Equations (4) and (5) fitted to the experimental data of broccoli by-products.

	Thawed by-products		Thawed by-products after cold-press dewatering	
	Test 1	Test 2	Test 1	Test 2
t_c [min]	$1.45{\cdot}10^2 \pm 23.03$ [a]	$1.40{\cdot}10^2 \pm 30.51$ [a]	$1.55{\cdot}10^2 \pm 11.71$ [a]	2.87 ± 136.32 [a]
$k_1 \left[\frac{\text{g desorbed water}}{\text{100 g dry matter}} \cdot \frac{1}{\text{min}}\right]$	2.64 ± 0.12 [a]	2.33 ± 0.13 [a]	1.39 ± 0.03 [a]	3.33 ± 5.35 [a]
$k_2 \left[\frac{\text{g desorbed water}}{\text{100 g dry matter}}\right]$	375.11 ± 48.24 [a]	416.23 ± 57.25 [a]	67.59 ± 14.09 [a]	263.63 ± 439.01 [a]
$\gamma \left[\frac{\text{kWh}}{\text{min}}\right]$	0.0163 ± 0.0001 [b]	$0.0173 \pm 5.79{\cdot}10^{-5}$ [a]	0.01754 ± 0.00011 [a]	0.0166 ± 0.0001 [b]
$\bar{E}$% Dehydration Kinetic	5.74 [a]	3.89 [b]	1.95 [c]	1.89 [d]
$\bar{E}$% Energy Consumption	10.51 [a]	6.10 [d]	9.83 [b]	9.59 [c]

Data are presented as mean ± standard deviation. In each row, different lowercase letters denote statistically significant differences ($p < 0.05$).

Table S2: Model parameters of Equations (4) and (5) fitted to the experimental data of asparagus by-products.

	Thawed by-products		Thawed by-products after cold-press dewatering	
	Test 1	Test 2	Test 1	Test 2
t_c [min]	$4.93 \cdot 10^{-5} \pm 435.82$ [a]	$1.39 \cdot 10^{-1} \pm 212.14$ [a]	$9.21 \cdot 10^{-5} \pm 1711.8$ [a]	$2.15 \cdot 10^{2} \pm 20.66$ [a]
$k_1 \left[\frac{\text{g desorbed water}}{\text{100 g dry matter}} \cdot \frac{1}{\text{min}}\right]$	7.11 ± 17.21 [a]	3.48 ± 253.23 [a]	3.63 ± 47.16 [a]	1.53 ± 0.0440 [a]
$k_2 \left[\frac{\text{g desorbed water}}{\text{100 g dry matter}}\right]$	1276.5 ± 3077.7 [a]	1015.3 ± 73860 [a]	477.87 ± 6213.2 [a]	114.72 ± 26.83 [a]
$\gamma \left[\frac{\text{kWh}}{\text{min}}\right]$	$0.0175 \pm 4.31 \cdot 10^{-5}$ [a]	$0.0172 \pm 2.98 \cdot 10^{-5}$ [b]	$0.0174 \pm 6.70 \cdot 10^{-5}$ [a,b]	$0.0174 \pm 6.42 \cdot 10^{-5}$ [a,b]
$\overline{E}$% Dehydration Kinetic	2.88 [d]	4.55 [a]	3.59 [c]	4.46 [b]
$\overline{E}$% Energy Consumption	4.64 [c]	2.97 [d]	6.64 [a]	6.29 [b]

Data are presented as mean ± standard deviation. In each row, different lowercase letters denote statistically significant differences ($p < 0.05$).

Table S3: Model parameters of Equations (4) and (5) fitted to the experimental data of artichoke by-products.

	Thawed whole Artichoke		Thawed whole Artichoke + Extractor	
	Test 1	Test 2	Test 1	Test 2
t_c [min]	0.00 [c]	162.46 ± 26.21 [a]	114.5 ± 12.3 [a,b]	107.0 ± 20.8 [b]
$k_1 \left[\frac{\text{g desorbed water}}{\text{100 g dry matter}} \cdot \frac{1}{\text{min}}\right]$	4.17 ± 0.195 [a]	2.08 ± 0.10 [b]	1.10 ± 0.03 [c]	1.260 ± 0.073 [c]
$k_2 \left[\frac{\text{g desorbed water}}{\text{100 g dry matter}}\right]$	625.1 ± 7.46 [a]	229.8 ± 44.4 [b]	53.99 ± 11.54 [c]	42.9 ± 21.4 [c]
$\gamma \left[\frac{\text{kWh}}{\text{min}}\right]$	$0.0167 \pm 5.50 \cdot 10^{-5}$ [c]	$0.0170 \pm 3.03 \cdot 10^{-5}$ [b]	$0.0172 \pm 7.17 \cdot 10^{-5}$ [a]	$0.0172 \pm 6.92 \cdot 10^{-5}$ [a]
$\bar{E}$% Dehydration Kinetic	3.96 [b]	4.50 [a]	2.21 [d]	3.86 [c]
$\bar{E}$% Energy Consumption	5.99 [b]	3.49 [d]	5.61 [c]	6.04 [a]

Data are presented as mean ± standard deviation. In each row, different lowercase letters denote statistically significant differences ($p < 0.05$).
